# Evaluation of portal venous system in post splenectomised beta-thalassemic children: A prospective study in a tertiary care hospital

**DOI:** 10.1016/j.amsu.2022.103565

**Published:** 2022-04-18

**Authors:** Shirish Silwal, Dinesh Prasad Koirala, K.M. Didarul Islam, Sanjeev Kharel, Sita Thapa, Subita Neupane

**Affiliations:** a Department of Pediatric Surgery, Kanti Children's Hospital, Kathmandu, Nepal; b Department of General Surgery, Pediatric Surgery Unit, Tribhuvan University Teaching Hospital, Institute of Medicine, Kathmandu, Nepal; c Department of Pediatric Surgery, Bangabandhu Sheikh Mujib Medical University, Dhaka Bangladesh; d Maharajgunj Medical Campus, Tribhuvan University, Institute of Medicine, Kathmandu, Nepal; e Kathmandu Medical College, Kathmandu University, Kathmandu, Nepal; f Department of General Practice and Emergency Medicine, National Academy of Health Science, Kathmandu, Nepal

**Keywords:** Thalassemia, β-Thalassemia, Portal venous system, Splenectomy, Portal vein thrombosis, Portal hypertension

## Abstract

**Background:**

Splenectomy is a palliative management technique in children with β-thalassemia. Portal thrombosis is the most dreaded complication after splenectomy that requires fast diagnosis, effective therapy, and good follow-up to prevent protal hypertension. Thus, there is the importance of constant evaluation of portal venous system through Color Doppler Ultrasound. This cohort study aimed to observe the changes in the portal venous system in post-splenectomised β-thalassemic children.

**Material and methods:**

This is a prospective observational cohort study carried out on all the pediatric patients who have undergone splenectomy in Bangabandhu Sheikh Mujib Medical University, Dhaka Bangladesh from 2017 to 2019 for β-thalassemia. The color doppler of the portal venous system was done within the 7th to 10th post-operative day and after 3 months. Outcomes like mean the caliber of the portal vein, mean velocity within the portal vein, and color Doppler findings like Portal Vein Thrombosis (PVT) and Pathological change in Mean Volume (PMV) were calculated and compared in two headings: pre-operative period and postoperative period (7–10 POD and 3 months) with the help of paired *t*-test.

**Results:**

Twenty-Eight β-thalassemia patients with a mean age of 10.43 ± 3.91 years planned to undergo splenectomy were included in our study. The pre-operative mean caliber and mean velocity of the portal vein were not statistically significant when compared after the postoperative period (7–10 POD and 3 months). But, continuous changes in portal vein were seen that could lead to normalization or pathological changes.

**Conclusion:**

There are physiological and pathological changes in portal vein following splenectomy that could lead to varied complications like portal vein thrombosis and portal hypertension. Color Doppler Ultrasound findings along with close follow-up help in minimizing the pathological changes and complications.

## Highlights

• Splenectomy is a palliative management technique in children with β-thalassemia.
• Portal vein thrombosis and portal hypertension are dreaded complications.

## 1 Background

Thalassemia is the name given to a globin gene disorder that results in a diminished rate of synthesis of one or more of the globin chains and consequently, a reduced rate of synthesis of the hemoglobin [1]. β-thalassemia syndromes are a group of hereditary blood disorders characterized by reduced or absent beta globin chain synthesis [2].

The management of β-thalassemia in this part of the world has been forced to focus more on conservative and palliative management rather than definite treatment. Conservative management includes life-long blood transfusion, iron chelating therapy, and management of associate complications improving their life expectancy rate [3]. Splenectomy as a palliative management, is indicated in those patients with annual blood transfusion requirement of more than 200–220 ml/kg/year, hypersplenism, symptomatic splenomegaly [4,5].

Portal vein thrombosis is one of the complications after splenectomy that requires fast diagnosis, effective therapy, and a good follow-up to prevent portal hypertension [6]. The incidence of portal venous thrombosis was 23–26% in post-splenectomised thalassemia patients [7]. This thrombosis can lead to varied complications such as mesenteric vein thrombosis, bowel ischemia, and hepatic dysfunction in the short-term and esophageal variceal bleeding from portal hypertension in the long-term period [8].

In various studies it has been estimated that the average time of development of thrombosis in post-operative splenectomy is 6.1 ± 3.5 days [9], within 2 weeks [[10], [11], [12], [13]], and in 7th and 30th postoperative day [14,15].

These splenectomised patients are at a hypercoagulable state due to diverse factors such as thrombophilia, increased platelet aggregation, formation of reactive O2 species, decreased levels of antithrombin III, protein C, and protein S, and deregulation of nitric oxide homeostasis, therefore these patients are at high risk of thrombosis [14,16,17].

Color Doppler ultrasound being noninvasive, portable, easily available with quick examination time is an investigation of choice for identifying portal vein thrombosis. It also provides information about the hemodynamic alterations in the hepatic portal system like pressure, direction, and velocity of flow, with a sensitivity of 85.7–89% and a specificity of 96–100% [[18], [19], [20]]. In the case of portal vein stenosis there is a high flow velocity, reduce diameter and area and in portal vein thrombosis, there is an absence flow, increase diameter, and area [21].

PHT (Portal hypertension) is defined as a pathologic increase of portal venous pressure that can be characterized by the hepatic vein pressure gradient greater than 5 mm Hg [22]. The physiological change such as dilatation of portal vein, decreased flow velocity and flow reversal are associated with PHT, therefore, the sensitivity of these parameters in the diagnosis of PHT is relatively high [21].

Thus, we aimed to assess portal vein thrombosis in a cohort of patients to prevent the risk of PHT through Color Doppler Ultrasound by comparison of outcomes like mean the caliber of the portal vein, mean velocity within a portal vein in the pre-operative and postoperative period (7–10 POD and 3 months). Similarly, we also assessed the normalization or pathological changes in both the pre-and post-operative period.

## 2 Materials and methods

Our study was a prospective cohort study conducted from 2017 to 2019 and was approved by the ethics committees of the Institutional Review Board (IRB) of Bangabandhu Sheikh Mujib medical University, Dhaka Bangladesh with unique identification approval number (BSMMU/2018/10352). The study was conducted in full conformance with principles of the Declaration of Helsinki. This study is reported according to STROCSS criteria [23].

**Inclusion and Exclusion Criteria:** The patients under the age of 18 years diagnosed with β-thalassemia and undergone splenectomy for prophylactic management were included in our study. Patients with useful data on Color Doppler Ultrasound findings done pre-operatively and post-operatively within 7th to 10th POD and 3 months were only included. While with β-thalassemia children managed conservatively and who lost on follow-up were excluded in our study.

### 2.1 Variables

The main outcomes needed for assessment pre-operatively and post-operatively are: mean caliber and mean velocity of the portal vein. The normal and pathological changes in portal vein seen in Color Doppler Ultrasound are collected. Demographic characteristics like mean age, age groups and sex ratio are also included.

### 2.2 Statistical analysis

Demographic characteristics, clinical parameters, and primary outcomes with pathological findings were reported using descriptive statistics. For the comparison of pre-operative and post-operative outcomes, paired sample *t*-test was with a p-value was calculated was used. The percentage change was calculated among the groups. The test was two-tailed and considered significant at p < 0.05. All the statistical analysis were performed using SPSS version 23.0.

## 3 Results

This study includes an age range of 5–18 years with a mean age of 10.43 ± 3.91 years. 14 children (50%) were in the age group between 5 years and 10 years. 12 (43%) children were in the group 10 years–15 years and only 2 were between 15 and 20 years. Out of 28 children, 16 were male and 12 were female giving rise to a male: female ratio of 1.3: 1. In percentages, males were 57.1% and females were 42.9% (Table 1).

**Table-1:** Demographic characteristics of the study subjects (n = 28).

Variables	Frequency (n)	Percentage (%)
Age group		
0–5 yrs	0	0
5–10 yrs	14	50
10–15 yrs	12	43
15–20 yrs	2	7
Total	28	100.0
Mean ± SD Range	10.43 ± 3.91 (5–18) years	
Sex		
Male	16	57.1
Female	12	42.9
Total	28	100.0
Male: Female ratio	1.3:1	

A data of mean caliber of portal vein of 28 with β-thalassemia patients enrolled in this study was compared in pre-operative period and post-operative period. Table 2 shows a comparison of preoperative mean caliber (8.01 ± 1.23) of the portal vein with the mean caliber of the portal vein in the 7th day (8.57 ± 1.89) and 3 months (8.12 ± 2.25) with a p-value 0.077 and 0.783 respectively, which is statistically not significant. Table 3 shows a comparison of preoperative mean velocity (16.12 ± 1.59) of the portal vein with a mean velocity of the portal vein in 7th day (16.51 ± 11.2) and 3 months (15.71 ± 3.65) with a p-value 0.85 and 0.542 respectively, which is statistically not significant and similarly, between mean velocity in 7th day and 3-month postoperative period were p-value 0.711. Table 4 shows color Doppler findings were normal preoperatively but on the 7th POD, there were 2 (7.1%) Portal vein thrombosis and 5(17.9%) pathological changes in mean velocity (PMV) among 2 portal vein thrombosis one changed to normal, other to PMV at 3 months and 3 PMV finding were normal at 3 months, 2 PMV persist whereas among 2 children those finding were normal in 7th day develop portal vein thrombosis and PMV.

**Table 2:** Comparison of caliber of portal vein preoperative and postoperative (n = 28).

Caliber	Mean ± SD (mm)	n	% Change	p-value
Caliber baseline	8.01 ± 1.23	28	7%	0.077 ^ns^
Caliber at 7th day	8.57 ± 1.89	28		
Caliber baseline	8.01 ± 1.23	28	1.37%	0.783^ns^
Caliber at 3-month day	8.12 ± 2.25	28		

Note: Paired *t*-test was used to see the level of significance, ns = not significant.

**Table-3:** Comparison of mean velocity preoperative and postoperative (n = 28).

Mean velocity	Mean ± SD (cm/sec)	n	% Change	p-value
Mean velocity baseline	16.12 ± 1.59	28	2.41%	0.85^ns^
Mean velocity at 7th day	16.51 ± 11.2	28		
Mean velocity baseline	16.12 ± 1.59	28	2.5%	0.542^ns^
Mean velocity at 3 months	15.71 ± 3.65	28		
Mean velocity at 7th day	16.51 ± 11.2	28	4.7%	0.711^ns^
Mean velocity at 3 months	15.71 ± 3.65	28		

Note: Paired *t*-test was used to see the level of significance, ns = not significant.

**Table-4:** Color Doppler findings in preoperative, 7th day and 3 months (n = 28).

Color Doppler findings	Preoperative n (%)	7^th^PODn (%)	3 months n (%)
Normal	28(100)	21(75)	23(82.1)
PVT	0	2(7.1)	1(3.6)
PMV	0	5(17.9)	4(14.9)
Total	28(100)	28(100)	28(100)

Note: PVT: Portal Vein Thrombosis, PMV: Pathological change in mean velocity.

## 4 Discussion

This prospective cohort study evaluated the predictors and outcomes to assess the increased risk of Portal Vein Thrombosis and PHT after splenectomy in β-thalassemic children. The cause for the risk of venous-thromboembolism in the splenoportal system after splenectomy is immediate reactive thrombocytosis and an increase in circulating microparticles [24].

After splenectomy in β-thalassemia as stated before, there may be a chance of pathological change in portal vein thus, an early indicator for change in portal venous system caliber and mean velocity were evaluated. While assessing portal vein caliber in the preoperative and postoperative period, there was a 7% change in the caliber at 7th postoperative day whereas caliber decreases to 1.37% change in 3 months postoperative period, which was found statistically not significant (P-value 0.077 and 0.783 respectively). Although caliber has a certain indicator for pathological change, it is very difficult and tedious to record the actual change in diameter of the portal vein in both the expiration and inspiration phase of breathing in the pediatric age group. And apart from it, there are fewer published research studies related to change in caliber in portal vein in the pediatric age group. In this study, while comparing the mean velocity of a pre-operative and post-operative period in the 7th postoperative day there is a 2.41% (p-value 0.85) increase in the mean velocity. But unexpectedly, there was a decrease in mean velocity by 2.5% (p-value 0.542) in the 3 months and while comparing mean velocity between the 7th postoperative day and 3 month post-operative period there was a 4.7% (p-value 0.711) decrease in mean velocity which was statistically not significant. It may probably due to variabilities in the pathology of portal vein as in portal vein thrombosis mean velocity is 0, in partial thrombosis, it can be higher and in portal hypertension mean velocity is less than 12 cm/s.

While comparing pathological changes in the portal venous system on the 7th day and 3 months, on the 7th postoperative day seven subjects developed some pathological changes in the portal vein. 2 subjects (7.1%) had portal vein thrombosis and 5 subjects (17.9%) had portal hypertension (PHT). Among the 2 subjects who had portal vein thrombosis, in 3 months follow up one subject resolved to normal and other changed into PHT and among 5 subjects with Portal Hypertension, 3 subjects resolved to normal while 2 subjects still had Portal hypertension and those subject with normal finding in 7th day had 1 PHT and 1 portal vein thrombosis in 3-month color Doppler. This finding concluded that there is a continuous change in the portal vein that could lead to normalization or pathological changes.

The median time of occurrence of portal vein thrombosis after splenectomy is reported between 5 and 12 days (average, 1 week), but it can occur as long as 99 days after the operation [25]. Similarly, a study by Alexakis et al. performed Doppler ultrasonography of the portal vein system on postoperative Day (POD) 7 and POD 30 or when indicated according to patients' symptoms [14]. Similar method was used in our study for 7th, 30th POD and after 3 months.

A study conducted among 70 β-thalassemic patients who underwent splenectomy, found only one case of the case (2.9%). While, compared with 35 patients who did not underwent splenectomy, significantly increase hematocrit, red blood cell indices, and platelet counts in patients undergoing splenectomy [26]. The study included 48 β-thalassemic patients undergoing splenectomy showed an incidence of portal vein thrombosis 8.3% (4 out of 48 patients) and spleen weight as an independent factor for the presence of PVT. The thrombosis resolution was observed after a median of 165 days [14].

Finally, a recent study of 55 patients with β-thalassemic who underwent splenectomy showed an incidence of PVT 5.5% (3 out of 55 patients) presenting with symptoms of unexplained pain of abdomen, anorexia, and fever within the first 2 months post-surgery. The time of presentation post-splenectomy was at 21, 26, 35 days respectively. Female gender, huge spleen, and postoperative thrombocytosis were also shown as risk factors for PVT. Doppler ultrasound findings also suggest the risk of PVT. Thus, antiplatelet prophylactic therapy should be initiated immediately in patients with risk of PVT [27].

Splenectomy is reserved for patients with symptoms like splenomegaly, increase blood transfusion requirement, and pancytopenia. The surgery should be considered weighing the potential benefit outweighing the risk like portal vein thrombosis [5].

There are several limitations to our study. First, the sample size was small because of the small study period. Second, there were some possibly unknown risk factors that we were unable to measure. Despite, these limitations, our study improves the better understanding of physiological and pathological changes in portal veins and risks for PVT.

## 5 Conclusion

There are physiological and pathological changes in portal vein following splenectomy that could lead to varied complications like portal vein thrombosis and portal hypertension. Color Doppler can be one of the diagnostic tools for identifying such changes, and prophylaxis with close follow-up can be considered to minimize the ongoing pathological changes in the portal venous system.

## Ethical approval

Study was approved by the ethics committees of the Institutional Review Board (IRB) of Bangabandhu Sheikh Mujib medical University, Dhaka Bangladesh.

## Sources of funding for your research

The authors declare that this study had no funding source.

## Author contribution

SS, DPK, KMDI: Initiated the research, wrote the research proposal, conducted the research, did data entry and analysis, and wrote the manuscript. SS, DPK: Involved in the write-up of the methodology of the proposal and research work. SST: Contributed in analysis of data. SK, ST, SN: Wrote and edited the manuscript. KMDI, DPK: Reviewed the manuscript. The authors read and approved the final manuscript.

## Declaration of competing interest

No potential conflict of interest relevant to this article was reported.

## Registration of research studies

1. Name of the registry: OSF Registers
2. Unique Identifying number or registration ID: osf.io/ps85e
3. Hyperlink to your specific registration (must be publicly accessible and will be checked): https://archive.org/details/osf-registrations-p9h3c-v1

## Provenance and peer review

Not commissioned, externally peer reviewed.

## Consent

Written informed consent was obtained from the patient's guardians for publication of this study and accompanying images.

## Abbreviations

PVT: Portal Vein Thrombosis
PMV: Pathological change in Mean Volume
POD: Post-Operative Day
PHT: Portal Hypertension

IRB- Institutional Review Board.

## References

[1] Bain B.J. (2006).

[2] Galanello R., Origa R. (2010). Beta-thalassemia. Orphanet J. Rare Dis..

[3] Bhosale M., Chandanwale A., Kinikar A., Singh D., Chaudhary R. (2015). Impact of splenectomy on quality of life of children with β-thalassemia. Int. J. Med. Publ. Health.

[4] Cappellini M.D. (2014). Guidelines for the Management of Transfusion Dependent Thalassaemia (TDT).

[5] Weledji E.P. (2014). Benefits and risks of splenectomy. Int. J. Surg..

[6] Skarsgard E., Doski J., Jaksic T. (1993). Thrombosis of the portal venous system after splenectomy for pediatric hematologic disease. J. Pediatr. Surg..

[7] Canatan D., Zorlu M., Bayir N. (2001). Thrombosis after splenectomy in patients with thalassemia. Turk. J. Haematol..

[8] Brink J.S., Brown A.K., Palmer B.A., Moir C., Rodeberg D.R. (2003). Portal vein thrombosis after laparoscopy-assisted splenectomy and cholecystectomy. J. Pediatr. Surg..

[9] Winslow E.R., Brunt L.M., Drebin J.A., Soper N.J., Klingensmith M.E. (2002). Portal vein thrombosis after splenectomy. Am. J. Surg..

[10] Hassn A.M., Al‐Fallouji M.A., Ouf T.I., Saad R. (2000). Portal vein thrombosis following splenectomy. Br. J. Surg..

[11] Ikeda M., Sekimoto M., Takiguchi S. (2005). High incidence of thrombosis of the portal venous system after laparoscopic splenectomy: a prospective study with contrast-enhanced CT scan. Ann. Surg..

[12] Petit P., Bret P.M., Atri M., Hreno A., Casola G., Gianfelice D. (1994). Splenic vein thrombosis after splenectomy: frequency and role of imaging. Radiology.

[13] Romano F., Caprotti R., Scaini A., Conti M., Scotti M., Colombo G., Uggeri F. (2006). Elective laparoscopic splenectomy and thrombosis of the spleno-portal axis: a prospective study with ecocolor doppler ultrasound. Surg. Laparosc. Endosc. Percutaneous Tech..

[14] Alexakis N., Dardamanis D., Albanopoulos K. (2013). Incidence, risk factors, and outcome of portal vein thrombosis after laparoscopic-assisted splenectomy in β-thalassemia patients: a prospective exploratory study. J. Laparoendosc. Adv. Surg. Tech..

[15] Chaffanjon P.C., Brichon P.Y., Ranchoup Y., Gressin R., Sotto J.J. (1998). Portal vein thrombosis following splenectomy for hematologic disease: prospective study with Doppler color flow imaging. World J. Surg..

[16] Cappellini M.D., Musallam K.M., Marcon A., Taher A.T. (2009). Coagulopathy in beta-thalassemia: current understanding and future perspectives. Mediterr. J. Hematol. Infect. Dis..

[17] Rosu A., Searpe C., Popescu M. (2012).

[18] Merritt C.R. (1987). Doppler color flow imaging. J. Clin. Ultrasound.

[19] Tudway D., Sangster G. (1986). Ultrasound diagnosis of portal vein thrombosis following splenectomy. Postgrad. Med..

[20] Vanwaeyenbergh J., Vierendeels T., Smekens L., Delvaux G., Willems G. (1989). Peroperative ultrasonography in the diagnosis of splenic vein thrombosis. Hepato-Gastroenterology.

[21] Ibinaiye P.O., Aiyekomogbon J.O., Tabari M.A., Chom N.D., Hamidu A.U., Yusuf R. (2015). Determination of normal portal vein parameters on triplex ultrasound scan among adults in Zaria, Nigeria. Sub-Saharan Afr. J. Med..

[22] Harkanyi Z. (2006). Pediatric portal hypertension. Ultrasound Clin..

[23] Mathew G., Agha R., Albrecht J., Goel P., Mukherjee I., Pai P., D'Cruz A.K., Nixon I.J., Roberto K., Enam S.A., Basu S. (2021 Dec 1). STROCSS 2021: strengthening the reporting of cohort, cross-sectional and case-control studies in surgery. Int. J. Surg. Open.

[24] Mohren M., Markmann I., Dworschak U. (2004). Thromboembolic complications after splenectomy for hematologic diseases. Am. J. Hematol..

[25] Krauth M.T., Lechner K., Neugebauer E.A., Pabinger I. (2008 Aug 1). The postoperative splenic/portal vein thrombosis after splenectomy and its prevention–an unresolved issue. Haematologica.

[26] Ammar S.A., Elsayh K.I., Zahran A.M., Embaby M. (2014). Splenectomy for patients with β-thalassemia major: long-term outcomes. Egypt. J. Surg..

[27] Zwain K.M., Al-Hakkak S.M., Al-Wadees A.A., Majeed Z.M. (Oct. 2019). Portal vein thrombosis post-splenectomy in thalassemia major patients. Int. J. Res. Pharm. Sci..

